# Phrenic nerve palsy during cryoballoon ablation of atrial fibrillation: a minor complication or a wolf in sheep's clothing? Insights on late arrhythmia recurrences from a propensity score-matched analysis

**DOI:** 10.3389/fcvm.2025.1650358

**Published:** 2025-09-29

**Authors:** Federico Cecchini, Giacomo Mugnai, Riccardo Maj, Andrea Rubboli, Alvise Del Monte, Domenico Giovanni Della Rocca, Alexandre Almorad, Juan Sieira, Alessio Marinelli, Alessandro Costa, Stefano Bonapace, Giulio Molon, Andrea Sarkozy, Gian Battista Chierchia, Carlo de Asmundis

**Affiliations:** ^1^ Division of Cardiology, Department of Emergency, Internal Medicine, and Cardiology, S. Maria delle Croci Hospital, Ravenna, Italy; ^2^ Heart Rhythm Management Center, Postgraduate Program in Cardiac Electrophysiology and Pacing, European Reference Networks Guard-Heart, Universitair Ziekenhuis Brussel - Vrije Universiteit Brussel, Jette, Belgium; ^3^ Electrophysiology and Cardiac Pacing, Division of Cardiology, Department of Medicine, Azienda Universitaria Ospedaliera Integrata Verona, Verona, Italy; ^4^ Department of Cardiology, IRCCS Sacro Cuore - Don Calabria, Negrar di Valpolicella, Italy

**Keywords:** phrenic nerve palsy, atrial fibrillation, cryoballoon ablation, pulmonary vein isolation, propensity score matching

## Abstract

**Background:**

Pulmonary vein isolation (PVI) has become the cornerstone of percutaneous atrial fibrillation (AF) treatment, and cryoballoon ablation (CB-A) has been shown to be non-inferior to radiofrequency (RF) ablation in terms of safety and efficacy. Hemi diaphragmatic paralysis, secondary to right phrenic nerve palsy (PNP), is the most common complication associated with cryoballoon ablation, occurring in 1.1%–6.2% of procedures. Although, in the literature, PNP appears to not jeopardize the acute electrical isolation of the right-sided pulmonary veins, its occurrence forces the electrophysiologist to immediately stop cryoenergy delivery, thus resulting in a shorter cryo-application time.

**Aims:**

The purpose of the analysis is to understand whether the occurrence of intraprocedural PNP can be related to an increased risk of developing atrial fibrillation/atrial flutter (AT/AF) recurrences during the follow-up.

**Methods and results:**

We retrospectively enrolled 116 consecutive patients who experienced PNP during PVI using the second-generation cryoballoon. This group was compared using 1:1 propensity score (PS) and caliper matching with a cohort of patients who did not present PNP. After the matching, 108 patients with CB-A related phrenic nerve palsy (PNP+ group) were analyzed and compared with 108 patients who did not experienced PNP (PNP- group). After a median follow-up of 19 [12.0, 31.0] months, the success rate was significantly lower in the PNP+ group compared to the control group (57.4% vs. 78.7%, Log-Rank *p* = 0.001). Patients with a history of PNP had an almost threefold higher risk of developing arrhythmia recurrences during follow-up compared to their counterparts (HR: 2.813, 95% CI: 1.470–5.385, *p* = 0.002). Patients who experienced PNP showed a trend toward a higher need for RF touch-up compared to the counterpart (*p* = 0.06). Left atrial diameter (Lad), need of cardioversion to restore sinus rhythm after PVI, together with PNP were found to be independent predictors of late arrhythmia relapses. No major complications occurred in the entire population.

**Conclusions:**

The present study showed that PNP, although typically reversible and without long-term sequelae, is associated with a significantly lower success rate of CB-A PVI and a higher risk of arrhythmia recurrences.

## 1 Introduction

Atrial fibrillation (AF) is the most common sustained cardiac arrhythmia in adults, with an estimated prevalence ranging from 2% to 4% in the general population (1).

Over the years, pulmonary vein isolation (PVI) using the second-generation cryoballoon (CB2, Arctic Front Advance, Medtronic Inc., Minneapolis, USA) has gained recognition as an effective treatment option for symptomatic AF (2–6), being non-inferior to Radiofrequency point-by-point catheter Ablation (RFCA) with respect to success and complication rates (7).

Hemi diaphragmatic paralysis secondary to right phrenic nerve palsy (PNP) is the most common CB-A related complication, with a reported incidence of 1.1%–6.2% (8–12). Histopathologically, it is characterized by axonal injury with Wallerian degeneration (13). In the vast majority of cases, immediate balloon deflation is the essential manoeuvre which causes rapid tissue rewarming and prevents the formation of a permanent lesion (14). As a matter of fact, PNP is most of the time transient and can last from a few seconds or minutes up to several months.

PNP is most of the times asymptomatic: a recent article from the Netherlands Heart Registration reported that among the 44 patients with follow-up data on PNP-related symptoms, 38 (86.4%) were free of PNP-related symptoms after a median follow-up of 184 (82–359) days (15).

Although in the literature PNP does not appear to jeopardize the acute electrical isolation of the right-sided pulmonary veins (16–18), its occurrence forces the electrophysiologist to interrupt the cryoenergy delivery, resulting in shorter cryo-application time. To date, data regarding PNP and its potential association with late arrhythmia recurrences are limited to a single study involving 23 patients with a mean follow-up of less than 12 months (19).

## 2 Methods

### 2.1 Inclusion and exclusion criteria

All patients with symptomatic, drug-refractory AF who underwent PVI using the CB2 at our EP centers (IRCSS Sacro Cuore, Negrar di Valpolicella, Italy; UZ Brussel-VUB, Brussels, Belgium) were retrospectively analyzed. Patients who experienced PNP (PNP+ group) during CB-A between October 2014 and May 2020 were selected and matched via propensity score with patients free from this complication (PNP- group).

Exclusion criteria included prior AF ablation, severe valvular or congenital heart disease, a left atrial diameter > 60 mm, intracardiac thrombi, uncontrolled heart failure, severe coronary artery stenosis.

Patient data referred to the Italian EP-center were extracted from the 1-STOP registry, a clinical data repository for an integrated network consortium of Italian cardiac hospitals. Conversely, data on patients treated at UZ Brussel were selected from a dataset collection approved by the local Ethical Committee. The study was conducted in accordance with the ethical standards outlined in the 1964 Declaration of Helsinki and its subsequent revisions.

### 2.2 Pre and post-procedural management

Each patient provided written informed consent prior to the procedure. Upon hospital admission, a blood sample was taken for standard hematological and biochemical testing. If transthoracic echocardiography (TTE) had not been performed within the last four weeks, a new TTE was repeated before the procedure. Direct oral anticoagulants (DOACs) were stopped 12–24 h before the procedure and resumed the same evening while patients on vitamin K antagonists continued anticoagulation therapy, targeting a pre-procedural INR of 1.5–2. Except for amiodarone, which was interrupted one month before CB-A, all other antiarrhythmic medications were discontinued 3–5 days prior to the ablation. All patients also underwent a pre-procedural heart computed tomography (HCT) to assess pulmonary vein anatomy and exclude significant coronary artery stenosis. In each patient, a transesophageal echocardiography (TEE) was performed after the induction of general anesthesia and prior to starting the procedure, to exclude the presence of intracardiac thrombi.

TTE was performed immediately after the ablation procedure and again 24 h later to check for any signs of pericardial effusion. Throughout the post-procedural hospital stay, patients were continuously monitored via ECG, and most were discharged the day after the procedure if their clinical status was stable. Before discharge, all patients underwent a clinical cardiological and vascular evaluation, to rule-out potential vascular complications.

In cases of PVI-related PNP, a chest x-ray during deep inspiration was performed the day after ablation to evaluate right hemidiaphragm contraction recovery. Anticoagulation therapy was recommended for at least two months post-ablation, after which it was adjusted based on the patient's prothrombotic risk (CHA2DS2-VASc score) and bleeding risk (HAS-BLED score) according to specific guidelines (1).

Antiarrhythmic drugs (AADs) that had previously been ineffective were continued for the first three months as per protocol. After this period, the decision to continue or discontinue the medications was left to the discretion of the patient's cardiologist.

### 2.3 Cryoballon PVI

The intraprocedural management has been described in detail in previous studies (20, 21). In summary, the procedures were conducted under general anesthesia, mechanical ventilation, and continuous invasive arterial pressure monitoring. To facilitate intubation, only short-acting paralytic agents were used to avoid compromising diaphragm contraction during right phrenic nerve stimulation. Unfractionated heparin (UFH) was administered as a bolus (100 IU/kg) immediately following the trans-septal puncture, with additional doses given every 30 min to maintain an activated clotting time (ACT) above 300 s.

An esophageal temperature probe (Sensitherm, St. Jude Medical) was introduced into the esophagus to monitor esophageal temperature during each cryo-application, which was promptly discontinued if the temperature dropped below 15°C (22). A 6 Fr decapolar catheter was initially advanced to the coronary sinus (CS) to provide fluoroscopic guidance for the trans-septal puncture. During ablation of the left-sided pulmonary veins (PVs), the decapolar catheter was moved from the CS to the right ventricular apex, and subsequently positioned in the superior vena cava (SVC) for phrenic nerve stimulation during ablation of the right-sided veins.

A single trans-septal puncture was performed via the femoral vein under fluoroscopic and transesophageal echocardiography (TEE) guidance using the modified Brockenbrough technique and an 8.5 Fr trans-septal long-sheath (SL1, St. Jude Medical Inc., St. Paul, USA). The trans-septal sheath was then exchanged over a guidewire with a 15 Fr steerable sheath (FlexCath Advance, Medtronic). A 28-mm second-generation cryoballoon catheter (Arctic Front Advance, Medtronic) and its accompanying inner lumen mapping catheter (ILMC) (Achieve, Medtronic) were introduced into the left atrium (LA) via the 15 Fr steerable long sheath. The ILMC was then positioned at each pulmonary vein ostium to record PV electrical activity and achieve real-time isolation (RTI). Starting from the left superior pulmonary vein (LSPV) and proceeding clockwise, the cryoballoon was inflated and carefully positioned at each PV ostium to achieve full occlusion. Optimal occlusion was defined as complete contrast retention within the vein and its branches, with no reflux into the left atrium (LA). During each cryo-application, if either electrical PV isolation or a temperature below −40°C was achieved within the first 60 s, the freeze was continued for either 180 or 240 s based on operator preference. If these criteria were not met, the application was aborted, and the cryoballoon repositioned for a better PV occlusion.

In the case of a left common ostium (LCO), the standard approach involved sequential cryo-applications to the superior and inferior venous branches, delivering one cryo-application per branch. The right middle PV (RMPV) was treated with cryo-application only if electrical isolation was not achieved during ablation of the larger inferior or superior right pulmonary veins (RIPV, RSPV).

During cryo-application to the right-sided PVs, continuous phrenic nerve stimulation was achieved by placing the decapolar catheter in the SVC. Pacing commenced only when the cryoballoon temperature reached −20°C to avoid balloon displacement due to diaphragmatic contractions early in the application. Stimulation was performed at high output/pulse width (20–24 mA, 1.0–2.0 msec) with a cycle length of 1,000–1,200 msec. Diaphragmatic contractions were assessed using both fluoroscopic and tactile feedback, and energy delivery was promptly stopped if diaphragmatic contraction diminished or disappeared. If PNP occurred, a waiting time of 20 min was adopted. During this timeframe, repeated assessments of diaphragmatic contractions were performed. If diaphragmatic contraction resumed and the PV was electrically silent, no further freezes were applied. However, when PV electrical activity was still present, another cryo-application was delivered.

In cases of PNP during RIPV ablation, treatment of the RSPV was performed only if the vein was electrically active and if the diaphragm contractility recovered. If the vein had potentials but PNP was still present, RSPV isolation was carried out using RF energy and a 4 mm irrigated-tip ablation catheter.

Following ablation of all PVs, the ILMC was used to check for early electrical reconnection. Entrance and exit block were confirmed for each vein. Atrial far-field activity was distinguished from PV potentials (PVPs) by pacing from the proximal or distal CS, left atrial appendage (LAA), or SVC while the ILMC was placed at the PV ostium. If reconnection was observed, a bonus freeze was applied. Successful PV isolation was defined as the absence of PV potentials or their dissociation from atrial activity.

If atrial fibrillation (AF) persisted after successful cryoballoon ablation (CB-A), electrical cardioversion (ECV) was performed to restore sinus rhythm (SR), and PV activity was reassessed during SR. Failure of CB-A was defined as the inability to isolate all PVs.

Procedure duration was defined as the time from the first groin puncture to the removal of all catheters from the patient.

### 2.4 Procedure-related complications

In line with the 2017 consensus statement on catheter ablation for atrial fibrillation (23), a complication was classified as procedure-related if it occurred during the ablation, during the hospital stay, or within 30 days after the procedure. A complication was considered “major” if it extended the hospital stay beyond 48 h, required an intervention or treatment, or caused permanent injury or death. Bleeding was recorded as a major complication only if it required blood transfusions or resulted in a hematocrit decrease of more than 20%.

PNP was defined as loss of right hemidiaphragm contraction during right-sided PVI, after confirming the pacing catheter stability.

### 2.5 Follow-up

Arrhythmia recurrence was defined as any episode of atrial fibrillation (AF) or atrial tachyarrhythmia (AT/AFL) lasting more than 30 s, detected via surface electrocardiogram (ECG) or rhythm monitoring device, following a 90-day blanking period (BP). Routine follow-up visits were conducted at 3, 6, and 12 months, and every 6 months thereafter, including anamnesis, clinical examination, surface ECG, and 24–48-hour Holter-ECG monitoring. Additionally, patients were instructed to contact their cardiologist or the nearest emergency department and undergo an extra Holter-ECG in the event of any symptoms suggestive of arrhythmia recurrence. For patients with pacemakers or ICDs, available device interrogation data were used to monitor arrhythmias. If symptoms arose in those with cardiac implantable electronic devices (CIEDs), updated data transmission or in-hospital device checks were strongly recommended.

In patients with intraprocedural PNP without signs of right hemidiaphragm contraction recovery the day after CB-A, a follow-up chest x-ray was scheduled after three months. If diaphragmatic impairment persisted, additional chest x-rays were scheduled after six and twelve months from ablation.

### 2.6 Repeat ablation procedure

Repeat ablation was offered to patients experiencing AF/AT recurrences despite optimized antiarrhythmic therapy. Repeat procedures were conducted using a 3D mapping system (CARTO, Biosense Webster© or EnSite NavX, Abbott©) in combination with a circular multielectrode mapping catheter (CMMC) and an open-irrigated, 4 mm-tip ablation catheter equipped with a contact force sensor. Radiofrequency (RF) was the only energy source used for re-do ablation, and our detailed procedural protocol has been described previously (24).

Briefly, following a double trans-septal puncture, a 3D electroanatomical map (EAM) of the left atrium (LA) was created, and PV electrical isolation was assessed using the CMMC. PV reconnection was defined as the re-establishment of electrical conduction between the LA and the PV that had been absent at the conclusion of the initial CB-A procedure. If reconnection was observed, RF energy was applied to re-isolate the vein, targeting gaps in conduction rather than ablating the whole quadrant.

In cases of durable PV isolation, additional substrate modification was performed for non-PV triggers and low-voltage areas. If a micro/macro-reentrant atrial tachycardia or flutter was present or induced, atrial activation map and pacing maneuvers (i.g. atrial entrainment) were used to understand the circuit characteristics before specific ablation. Point-by-point RF ablation was performed in a power-controlled mode, aiming for contact forces between 5 and 20 grams, with a maximum temperature setting of 45°C. A power limit of 35W was used in all areas except for the posterior atrial wall, where 30 W was applied. After a waiting period of at least 20 min following the final RF application, pacing maneuvers and 3D electroanatomical activation mapping were utilized to confirm bidirectional block along ablation lines and PV antra.

### 2.7 Study endpoints

The primary endpoint of the study was to assess any documented recurrence of AT/AF occurring after the BP and lasting longer than 30 s in patients with intraprocedural PNP (PNP+) and in the control group (PNP−).

Secondary endpoints included the identification of potential predictors for arrhythmia recurrence and phrenic nerve palsy.

### 2.8 Statistical analysis

A 1:1 propensity score (PS) matching was performed to compare patients who experienced PNP (PNP+ group) with those who did not (PNP- group). The propensity score matching was carried out including “age”, “gender”, “type of AF”, “arterial hypertension” (AHT), “diabetes mellitus” (DM), “LA diameter” (LAd), “heart failure” (CHF) in the logistic regression model (21, 25). The logistic model generated a propensity score (PS) for each observation, calculated as the predicted probability of experiencing PNP based on the aforementioned covariates. A caliper of 0.05 was set to automatically match the propensity scores, with patients from the control cohort paired with their nearest PS counterparts in the PNP+ group to ensure a balanced comparison between the two populations. The quality of the propensity score matching was assessed using Standardized mean difference (SMD), Love Plot and Variance Ratio as balance diagnostics (26).

Categorical variables are expressed as absolute frequencies and percentages. Continuous variables are presented as mean ± standard deviation or, for non-normally distributed data, with median and interquartile range (IQR). Normality was tested using the Shapiro–Wilk test.

Categorical variables were compared using the Chi-squared and Fisher's exact tests while the Student's t-test for independent samples or the Mann–Whitney tests were used for continuous data comparison. Cox regression was used to analyse the association between PNP and the outcome of AT/AF recurrence. The Kaplan–Meier survival function was instead used to graphically display arrhythmia relapses in both groups over time and a P log-rank was calculated.

The association between baseline/procedural characteristics and AT/AF recurrences was initially assessed by using univariate Cox regression analysis. Variables with a *p*-value < 0.1 in the univariate Cox regression analysis were included for the multivariate model. After performing the multivariate Cox regression analysis, the proportional hazards assumption was evaluated using the Schoenfeld residuals test. Additionally, the discriminative ability of the model was assessed by calculating Harrell's C-index.

Binary logistic regression was conducted to analyze the association between baseline/procedural characteristics and phrenic nerve palsy. Variables that appeared to be mildly linked with the outcome at the univariate analysis (*p*-value < 0.20) were included in the multivariate binary logistic model.

Statistical analyses was carried out using R Studio (R Development Core Team) and SPSS Statistics, version 29.0 (IBM Corp., Armonk, NY, USA). Statistical significance was defined as two-sided *p*-value of <0.05.

## 3 Results

### 3.1 Matching and population characteristics

A total of 116 patients with CB-A-related PNP were evaluated. After the propensity score matching with a 0.05 caliper, 108 patients who experienced PNP (PNP+ group) were matched and analyzed alongside 108 patients who did not develop this complication (PNP- group). While the instruments used to assess the quality of PSM are displayed in Supplementary File 1, baseline characteristics of the populations are summarized in Table 1.

**Table 1:** Baseline patient characteristics.

Patient characteristics	Total (*n*. 216)	PNP- (*n*. 108)	PNP+ (*n*. 108)	*P* value
Age (years)	64.0 [54.0, 71,0]	64.0 [57.0, 70.0]	64.0 [53.0, 71.0]	0.72
BMI (Kg/m^2^)	26.3 ± 4.2	26.4 ± 4.1	26.2 ± 4.2	0.72
Male gender, *n* (%)	121 (53.5)	59 (54.6)	62 (57.4)	0.78
Paroxysmal AF, *n* (%)	157 (72.7)	79 (73.2)	78 (72.2)	0.99
Persistent AF, *n* (%)	59 (30.5)	29 (26.8)	30 (27.8)	0.99
AF duration (months)	13.0 [5.0, 48.0]	12.0 [5.0, 60.0]	14.0 [4.0, 40.0]	0.94
LA diameter (mm)	42.3 [37.0, 47.0]	42.3 [37.0, 46.1]	42.3 [37.0, 47.0]	0.97
LVEF (%)	60.0 [56.0, 60.0]	60.0 [55.0, 60.0]	60.0 [60.0, 60.0]	0.16
Heart failure, *n* (%)	18 (8.3)	10 (9.3)	8 (7.4)	0.81
Dilated cardiomyopathy, *n* (%)	1 (0.5)	0 (0.0)	1 (0.9)	0.99
Coronary artery disease, *n* (%)	24 (11.1)	15 (13.9)	9 (8.3)	0.19
Arterial hypertension, *n* (%)	107 (49.5)	54 (50.0)	53 (49.1)	0.99
Diabetes mellitus, *n* (%)	14 (6.5)	7 (6.5)	7 (6.5)	0.99
COPD, *n* (%)	14 (6.5)	6 (9.2)	8 (10.0)	0.99
CKD, *n* (%)	20 (9.3)	8 (7.4)	12 (11.1)	0.48
Prior stroke/TIA, *n* (%)	14 (6.5)	7 (6.5)	7 (6.5)	0.99
PM, *n* (%)	9 (5.1)	5 (4.6)	4 (3.7)	0.47
ICD, *n* (%)	8 (4.6)	5 (4.6)	3 (2.8)	0.71
CHA_2_DS_2_VASc score	2.0 [1.0, 3.0]	2 [1.0, 3.0]	2 [1.0, 3.0]	0.53
N° of ECV before PVI, *n* (%)	0.0 [0.0, 1.0]	0 [0.0, 1.0]	1 [0.0, 1.0]	0.28
N° of AADs tested, *n* (%)	1.0 [1.0, 2.0]	2 [1.0, 2.0]	1 [1.0, 2.0]	0.66
Ic class AADs, *n* (%)	73 (33.8)	40 (37.0)	33 (30.6)	0.39
Beta blockers, *n* (%)	103 (47.7)	52 (48.2)	51 (47.2)	0.99
Amiodarone, *n* (%)	19 (8.8)	10 (9.2)	9 (8.3)	0.99
Sotalol, *n* (%)	3 (1.4)	2 (1.9)	1 (0.9)	0.70
Ca^2+^ - Antagonists, *n* (%)	31 (14.4)	14 (13.0)	17 (15.7)	0.56

Continuous data are summarized as mean ± standard deviation or as median [25th and 75th percentiles]. Categorical data are presented as number and percentages (%). *P* value refers to data comparison between the PNP+ cohort and the control group (PNP-). BMI, body mass index; AF, atrial fibrillation; LA, left atrium; LVEF, left ventricular ejection fraction; COPD, chronic obstructive pulmonary disease; CKD, chronic kidney disease; eGFR, estimated glomerular filtration rate (according to the Cockcroft-Gault formula); TIA, transient ischemic attack; PM, pacemaker; ICD, implantable defibrillator; ECV, electrical cardioversion; CHA2DS2_VASc_, congestive heart failure; hypertension, age, diabetes mellitus; stroke/TIA; vascular arterial disease; sex category (female); AAD, antiarrhythmic drugs.

In PNP+ group the median age was 64.0 [53.0, 71.0] years, while the median age for the control group was 64.0 [57.0, 70.0] years (*p* = 0.72).

As result of the matching, values distribution in variables such as AF duration, Age, Gender, BMI, AHT, DM, LAd and HF were very similar in both population (*P* ranging from 0.72 to 0.99).

Globally, 157 (72.7%) patients were affected by paroxysmal AF (PAF): 79 (73.2%) patients in the control group and 78 (72.2%) in the PNP+ group (*p* = 0.99).

CHA2DS2VASc score was on average low [2.0 (1.0, 3.0)] and not different between the groups (*p* = 0.53), reflecting the low presence of comorbidities in the overall population.

The vast majority of patients had a preserved kidney function with eGFR > 60 ml/min: as a matter of fact, only 8 (7.4%) subjects in the PNP- cohort and 12 (11.1%) in the PNP+ group were affected by CKD (*p* = 0.48).

Left ventricular ejection fraction (LVEF) did not significantly differ between the two populations [60.0% (55.0, 60.0) vs. 60.0% (60.0, 60.0), *p* = 0.16]; similarly the number of patients wearing cardiac implantable devices (CIEDs) was comparable between the PNP+ and PNP- group [7 (6.5%) vs. 10 (9.2%) patients, *p* = 0.45].

Regarding antiarrhythmic therapy, the median number of drugs tested prior to CB-A was similar between groups: 2 [1.0–2.0] in the control group and 1 [1.0–2.0] in the PNP+ group (*p* = 0.66).

### 3.2 Procedural characteristics

Data about the main procedural characteristics are listed in Table 2*.*

**Table 2:** Procedural data.

Ablation characteristics & parameters	PNP-	PNP+	*P* value
Procedure duration, (min)	75.0 [60.0, 100.0]	78 [69.0, 102.0]	0.007
Fluoroscopic exposure, (min)	11.0 [8.0, 16.5]	12.0 [9.0, 17.0]	0.30
PVs anatomical variants, *n* (%)	16 (14.8)	14 (13.0)	0.69
Left common ostium	8 (7.4)	7 (6.5)	0.99
Right middle pulmonary vein	6 (5.6)	6 (5.6)	0.99
Number of PVs isolated, *n* (%)	430/430 (100%)	430/430 (100%)	0.99
AF at the beginning of CB-A, *n* (%)	19 (17.6)	10 (9.3)	0.11
SR restoration during PV freeze, *n* (%)	3 (2.8)	1 (0.9)	0.62
ECV at the end, *n* (%)	25 (23.1)	22 (20.4)	0.74
LSPV			
Number of freezes	1.0 [1.0, 1.0]	1.0 [1.0, 1.0]	0.26
Time to isolation (sec)	38.0 [30.8–45.3]	37.0 [30.0–50.0]	0.87
Temperature at 60 sec (°C)	−42.0 [−44.5, −39.5]	−42.0 [−42.25, −40.0]	0.48
Minimum temperature (°C)	−50.5 ± 5.7	−51.1 ± 5.3	0.89
Total freeze duration (sec)	180.0 [180.0, 240.0]	180.0 [180.0, 240.0]	0.72
LIPV			
Number of freezes	1.0 [1.0, 2.0]	1.0 [1.0, 2.0]	0.95
Time to isolation (sec)	34.0 [26.0–40.0]	36.5 [24.3–44.8]	0.40
Temperature at 60 sec (°C)	−40.0 [−43.5, −37.0]	−40.5 [−43.0, −38.0]	0.79
Minimum temperature (°C)	−46.0 ± 5.6	−46.0 ± 5.7	0.35
Total freeze duration (sec)	180.0 [180.0, 360.0]	180.0 [180.0, 360]	0.61
LCO			
Number of freezes	2.0 [2.0, 2.0]	2.0 [2.0, 2.0]	0.99
Time to isolation (sec)	40.0 [36.5, 46.0]	46.0 [39.8, 52.0]	0.17
Temperature at 60 sec (°C)	−42.0 [−45.3, −40.3]	−45.0 [−51.5, −41.5]	0.41
Minimum temperature (°C)	−48.5 [−50.0, −47.3]	−50.0 [−60.0, −48.5]	0.41
Total freeze duration (sec)	360.0 [360.0, 360.0]	360.0 [360.0, 360.0]	0.99
RIPV			
Number of freezes	1.0 [1.0, 1.0]	1.0 [1.0, 1.0]	0.56
Time to isolation (sec)	40.0 [33.8, 51.3]	37.0 [30.5, 44.8]	0.12
Temperature at 60 sec (°C)	−40.0 [−36.0, −44.5]	−43.0 [−40.0, −45.0]	**0**.**04**
Minimum temperature (°C)	−50.5 ± 5.2	−50.6 ± 5.7	0.59
Total freeze duration (sec)	180.0 [180.0, 240.0]	134.0 [122.8, 150.8]	**<0**.**001**
RSPV			
Number of freezes	1.0 [1.0, 1.0]	1.0 [1.0, 1.0]	0.72
Time to isolation (sec)	39.0 [30.3–45.0]	34.0 [27.0, 39.00]	0.11
Temperature at 60 sec (°C)	−44.0 [−45.5, −40.0]	−45.0 [−47.0, −42.0]	**0**.**03**
Minimum temperature (°C)	−50.0 ± 15.3	−50.7 ± 6.6	0.34
Total freeze duration (sec)	180 [180.0, 240.0]	133.5 [118.0, 149.5]	**<0**.**001**
RF touch-up, *n* (%)	0 (0.0)	5 (4.6)	0.06

Continuous procedural data are expressed as mean and SD if normally distributed, while with median [25th and 75th percentiles] in case of non-normal distribution. Categorical data are expressed as *n* (%). PV, pulmonary vein; CB-A, cryoballoon ablation; AF, atrial fibrillation; SR, sinus rhythm; ECV, electrical cardioversion; LSPV, left superior pulmonary vein; LIPV, left inferior pulmonary vein; LCO, left common ostium; RIPV, right inferior pulmonary vein; RSPV, right superior pulmonary vein; RF, radiofrequency. *P* values refer to data comparison between groups. PNP+ and PNP− refer, respectively, to patients who experienced and those who did not experience phrenic nerve palsy.

Procedure duration was longer in the PNP+ group compared to the control group [78.0 (69.0, 102.0) vs. 75.0 (60.0, 100.0) minutes, *p* = 0.007] while the fluoroscopic exposure was similar [12.0 (9.0, 17.0) vs. 11.0 (8.0, 16.5) minutes, *p* = 0.30].

No significant difference was found in the mean/median minimum temperature reached during freezes for each PV (*p*-value ranging between 0.34 and 0.89).

Total freeze duration was not different for left superior PV (LSPV), left inferior PV (LIPV), and left common ostium (LCO) (*p* = 0.72; *p* = 0.61; *p* = 0.99) but was significantly shorter in the PNP+ group compared to the control cohort, for both RIPV [134.0 (122.75, 150.75) sec vs. 180.0 (180.0, 240.0) sec, *p* < 0.001] and RSPV [133.5 (118.0, 149.5) sec vs. 180 (180.0, 240.0) sec, *p* < 0.001]. In the same way, also the temperatures reached after one minute of freeze were lower in the PNP+ group for RIPV [−43.0 (−45.0, −40.0)°C vs. −40.0 (−44.5, −36.0)°C], *p* = 0.04) and RSPV [−44.0 (−45.5, −40.0)°C vs. −45.0 (−47.0, −42.0)°C, *p* = 0.03], but similar for LSPV, LIPV and LCO (*p*-value ranging between 0.41 and 0.79). Time to isolation (TTI) proved to be similar for all pulmonary veins; a trend towards lower TTIs in the PNP+ group was present for RSPV and RIPV even if it did not reach statistical significance (*p* = 0.11 and 0.12, respectively).

At the beginning of the CB-A procedure, atrial fibrillation (AF) was present in 19 patients (17.6%) in the PNP- group compared to 10 patients (9.3%) in the PNP+ group (*p* = 0.11); after PVI, electrical cardioversion was performed in 25 patients (23.1%) of the PNP- group and in 22 patients (20.4%) from the PNP+ group (*p* = 0.74). Complete pulmonary vein isolation was achieved in 211/216 (97.7%) patients (425/430 veins).

In all cases of PNP during RSPV ablation, electrical isolation of the vein had either already been achieved or no baseline pulmonary vein signals were detectable at baseline. A similar situation was observed in RIPV cases where cryoapplication was prematurely interrupted due to PNP: although a shorter ablation, RIPV isolation was never compromised.

Since RSPV was the last vein treated according to our protocol, a complete PVI of all the veins was always possible even in cases of PNP during RSPV ablation. Conversely, in 5 out of 38 cases (13.2%) of PNP occurring during RIPV ablation, the RSPV remained electrically active (*P* < 0.01).

Pulmonary vein anatomical variants were observed in 14 patients (13.0%) in the PNP+ group and in 16 patients in the control group (*p* = 0.69); LCO was the commonest PV variant, being present in 15 over 216 patients (6.9%).

### 3.3 Procedure-related complications

No major complications occurred in the entire population.

Mild pericardial effusion was observed in only one patient of the PNP+ group (*p* = 0.99) which did not necessitate pericardiocentesis. Groin hematoma was the only vascular complication and occurred in a total of 4 (1.9%) patients, 3 (2.8%) for the PNP+ group and 1 (0.9%) for the control cohort (*p* = 0.62). In all the cases the hematoma did not require a surgical intervention, blood transfusion or prolonged hospitalization for more than 48 h.

Among the PNP cases, paralysis during RSPV ablation was more common, with 70 cases (64.8%) compared to 38 cases (35.2%) during RIPV ablation (*p* < 0.001).

30/38 (78.9%) PNPs during RIPV application recovered early during the procedure itself and therefore RSPV isolation was successfully performed without complications. Among the 8 remaining patients, RSPV was not treated in 3 cases because of absence of PV activity. In five cases, the use of the RF catheter was necessary to achieve the vein isolation (*p* = 0.06). Among RSPV-related PNP, in 30 cases (42.9%), recovery of hemidiaphragm contraction occurred during the procedure itself (*p* < 0.01).

In 48 patients (44.4%) PNP lasted longer than the procedure: in 10 cases recovery occurred during hospitalization. After 6 months three patients (2.8%) still had proof of PNP (two patients with RSPV-related PNP and one with PNP secondary to RIPV freeze, *p* = 0.9). After 12 months, 107/108 patients were healed from any PNP: the only patient with persistent PNP, which underwent pulmonary lobectomy after a chest trauma, had symptomatic effort dyspnea.

### 3.4 Follow-up

#### 3.4.1 Efficacy

**Figure 1:**
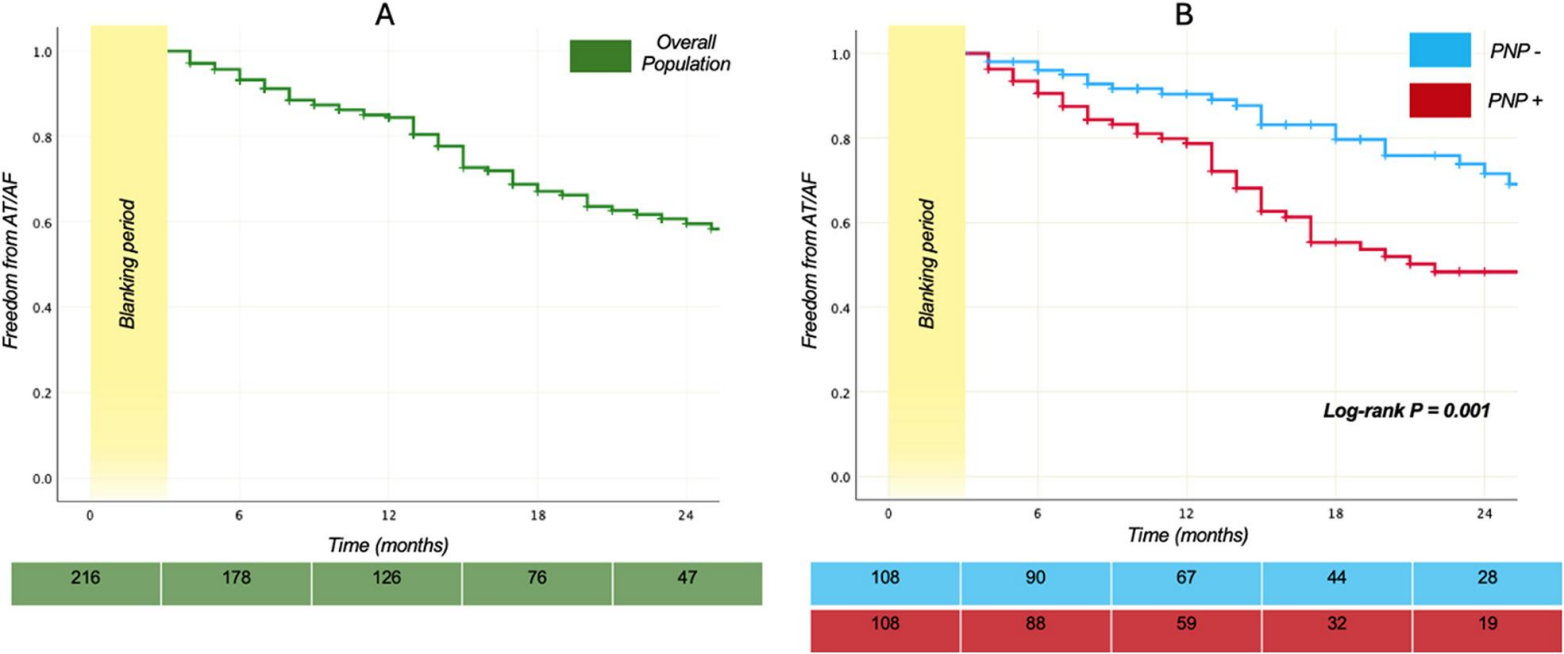
Survival plots. Kaplan–Meier curves demonstrating the relative proportion of patients without AT/AF recurrences following index PVI using the second-generation 28-mm cryoballoon. In the **(A)** panel the plot displays the proportion of the 216 patients who are free from arrhythmia recurrences over time, while the **(B)** panel shows a comparison of the survival curves in PNP+ and PNP- groups. Log-rank P between the groups was 0.001. The green and light blue - red table reports the number at risk for the overall population, the PNP- and the PNP+ group.

The Kaplan–Meier plot (Figures 1A,B) illustrates the proportion of patients without documented AT/AF recurrences following the index CB-A PVI procedure.

A median follow up of 19 months [12–31] was similar in both groups (*p* = 0.80). Overall, arrhythmia recurred in 69 of 216 patients (31.9%). Recurrence was significantly more common among patients who developed PNP during the procedure: 46 of 108 (42.6%) vs. 23 of 108 (21.3%) without PNP (log rank *p* = 0.001). Patients with PNP therefore had an unadjusted hazard of AT/AF recurrence more than twice that of controls [unadjusted HR 2.219, 95% CI (1.341, 3.670), *p* = 0.002].

Restricted mean survival time (RMST) over the first 24 months, was significantly shorter for the PNP+ group [18.02, CI (16.60, 19.45) months] compared to the control cohort [21.31, CI (16.17, 22.45) months], with a Diff RMST = 3.29 CI [−5.11, −1.46] months, *p* < 0.001.

After 12 months, 80.5% among the 216 patients were free from arrhythmia recurrences; this percentage progressively reduced over time until reaching a value of 59.5% two years later from CB-A (Figure 1A).

AT/AF relapses during BP had a trend to be more frequent in patients who experienced PNP (*p* = 0.052) compared to the counterpart.

The proportion of patients without AADs was similar in both groups (52.4% for PNP- and 47% for PNP +, *p* = 0.533); in the same way the need of ECV to restore SR during the follow-up was similar (6 (5.6%) in PNP- vs. 11 (10.2%) in PNP+, *P* = 0.21)).

For three patients (1.4%), a heart-rate control strategy was embraced and no further attempts of restoring SR were performed. All these patients experienced phrenic nerve palsy during the procedure. No strokes/TIA or deaths occurred during the follow-up**.**

#### 3.4.2 Repeat procedures

Globally, 42 patients (19.4%) underwent at least one repeat ablation. Of these, 37 (88.1%) patients underwent only one procedure, while 5 (12.9%) patients were scheduled also for a second re-do ablation. We did not report or speculate on the outcomes and characteristics of second redo ablations as it lies beyond this study's scope.

The median time to first re-do was 13 [7, 20] months and was similar in both groups (*p* = 0.47). At the time of the first re-do procedure, 23 (54.8%) patients were in SR, 14 (33.3%) were in AF and 5 (11.9%) in atrial tachycardia or atrial flutter (AT/AFl).

Re-do procedures were more common in the PNP+ group compared with the PNP- cohort (29 (25.9%) vs. 13 (12.0%), *p* = 0.006. PNP+ patients had an OR of 2.68 CI [1.13–5.51] to undergo a redo procedure compared with the control group.

Among a total number of 167 PVs, 67 (40.1%) appeared to be reconnected. The number of PVs reconnected was significantly higher in patients who experienced PNP compared to patients who did not [1.0 (1.0; 1.0) vs. 2.0 (1.0; 2.0) respectively; *p* = 0.003].

In 29 (69%) out of the 42 cases of first re-do ablation, only PV re-isolation was performed and while with regard to left-sided veins, the rate of reconnection was similar between the groups (*p* = 0.51), for the right-sided PVs, electrical reconnection was more common in the PNP+ group compared to the PNP- cohort [27 (93.1%) vs. 8 (61.5%), *p* = 0.02]: more specifically, PNP+ patients were over 8 times more likely to have a right-sided PV reconnection compared to the PNP- group [OR 8.44 CI (1.37; 52.06)].

Globally, LSPV was reconnected in 14/37 patients, LIPV in 9/37 patients, LCO in 3/6 patients, RSPV in 20/42 patients, RIPV in 18/42 patients, RMPV in 3 patients. For 11/42 (31.0%) patients, PV re-isolation plus substrate modification was necessary while in the remaining 2/42 (4.7%) subjects only substrate modification was performed since all the PVs were found to be isolated. Substrate modification consisted of CTI ablation in 7/13 out of cases, posterior mitral isthmus line in 3/13, complex fragmented electrogram (CFAEs) ablation in 5/13 cases, focal AT in 2/13 cases and AVNRT, left atrial posterior wall isolation (LAPWI) in one case. The proportion of arrhythmia triggers originating outside the pulmonary veins was not significantly different between the groups (*p* = 0.28).

#### 3.4.3 Predictors of AT/AF recurrence

Data about univariate Cox regression analysis are summarized in Supplementary File 2*.*

After the univariate Cox regression analysis, “Left atrial diameter” [*p* = 0.003, CI (1.02–1.09)], “CKD” [*p* = 0.02, CI (1.11–3.87)], “creatinine” [*p* = 0.02, CI (1.47–65.93)], “Need of ECV at the end of CB-A to restore SR” [*p* = 0.02, CI (1.11–3.15)], “PNP” [*p* = 0.002, CI (1.34–3.67)], “RIPV total freeze duration” [*p* = 0.03, CI (0.99–1.00)], “RSPV total freeze duration” [*p* = 0.003, CI (0.99–1.00)], “LIPV total n. of freezes” [*p* = 0.08, CI (0.33–1.07)], resulted as significant predictors of AT/AF recurrence with a *p* < 0.1.

The 0.1 threshold was chosen because the total number of events was 69, limiting the number of variables that could be included in the multivariate analysis to avoid overfitting. Using a less stringent threshold, such as *p* < 0.2, would have resulted in the inclusion of too many variables, compromising the reliability of the model.

Since the collinearity between RSPV PNP and RSPV freeze duration, between RIPV PNP and RIPV freeze duration, only PNP was maintained in the Cox proportional hazard model (basically, phrenic palsy induces the operator to immediately stop cryoenergy delivery and therefore to cause a shortening of the normal freeze duration).

A multivariable Cox Regression analysis (MCRA) was performed with the aforementioned variables with *p* < 0.1. A larger LA diameter [HR = 1.049, 95% CI (1.010, 1.090), *p* = 0.014], need of ECV to restore SR after PVI [HR = 1.933, 95% CI (1.026, 3.641), *p* = 0.041], and phrenic nerve palsy [HR = 2.813, 95% CI (1.470, 5.385), *p* = 0.002] were all significant independent predictors of AT/AF recurrence. The multivariable model is displayed in Table 3 while Harrell's C-index and Schoenfield Tests on the model are shown in Supplementary File 2 - Table 3*.*

**Table 3:** Multivariable regression model.

Variable	*P*-value	95% Confidence interval	Hazard ratio
Total number of LIPV freezes	0.796	0.429–1.477	0.796
Left atrial diameter	**0** **.** **014**	**1.010**–**1.090**	**1**.**049**
Chronic kidney disease	0.195	0.788–3.206	1.590
Need of ECV after PVI	**0**.**041**	**1.026**–**3.641**	**1**.**933**
Phrenic nerve palsy	**0**.**002**	**1.470**–**5.385**	**2**.**813**

The table presents the results of the multivariable Cox regression analysis aimed at identifying independent baseline and procedural characteristics associated with AT/AF recurrences after cryoballoon ablation. LIPV, left inferior pulmonary vein; ECV, electrical cardioversion; PVI, pulmonary vein isolation.

#### 3.4.4 Predictors of PNP

Binary logistic regression was applied to identify potential predictors of phrenic nerve palsy between demographic, clinical and procedural variables. No independent predictors were identified for PNP occurring during cryoablation of RIPV or RSPV. Data related to this analysis are reported in Supplementary File 3*.*

## 4 Discussion

Although described after RF, phased RF, Laser or thoracoscopic ablation, PNP is significantly more frequent during CB-A and may be still considered as the Achilles heel of this procedure (15, 27–29), with an incidence rate ranging between 3.5%–10.8% in the literature (30).

Historically, CB-A related PNP is considered a minor complication because it is virtually always asymptomatic and occurring late during the right-sided veins freeze, it almost never compromise the acute electrical isolation of these structures (16, 18, 20, 28, 31). The worldwide YETI registry described a 53.9% of PNP recovery during the procedure itself (11). For instance, the vast majority of patients with cryoballoon-induced hemidiaphragmatic paralysis recover within minutes up to 12 months, with a 0.1%–0.6% of patients having a paralysis lasting more than one year (31). Our findings are in line with the aforementioned ones: we described a intraprocedural recovery of 55.6% and a persistence on PN impairment of 0.9% after one year from CB-A.

All these data find their counterpart in animal studies that have shown how histopathologically, the presence of cold-induced axonal damage through Wallerian degeneration, is most of the times accompanied by islands of neuroregeneration (13).

To the best of our knowledge this is the only study comparing patients who developed PNP during CB-A with a propensity score-matched cohort of subjects who did not. Salient results include the following: (1) Patients who experienced PNP during CB-A had a higher rate of arrhythmia relapses than the counterpart; (2) PNP is an independent predictor of AT/AF recurrences during follow-up.

In our study, patients with procedural PNP were almost three times as likely to experience arrhythmia recurrence during follow-up compared to the counterpart [adjusted HR: 2.813, CI (1.470–5.385), *p* = 0.002]. This fact can be explained by the significantly shorter freeze duration in the PNP+ group.

With the advent of the second generation cryoballoon, efficacy of CB-A increased but the incidence of right PNP remained non-negligible (32); therefore the dosing of cryoenergy became of critical importance.

The possibility of shortening the freeze application from 240 s to 180 s was supported by Ciconte and colleagues (33). The study, comparing the efficacy of a single 3-minutes approach vs. the standard 4-minute plus bonus-freeze, demonstrated a similar 2-years freedom from AT/AF recurrences among the 160 patients enrolled.

This data prompted electrophysiologists to investigate the extent to which cryoapplication duration could be shortened without compromising efficacy, in order to reduce procedure time and minimize complications.

Bianchini et al. reported a higher rate of non-sustained PVI after a single 120-second application while, a 120 s × 2 protocol reduced CB-A related complications and provided the same acute procedural efficacy as the standard 240-sec (34).

These data are in line with those reported in the CIRCA-DOSE trial (35, 36) where the authors found no significant difference in freedom from atrial tachyarrhythmias during one year of continuous invasive rhythm monitoring following the PVI procedure between patients randomized to contact force-guided RF ablation and two different CB-A protocols: cryoablation of 4 min vs. cryoablation of 2 min, both followed by a single additional application of the same length after the rewarming phase.

A further reduction in freeze duration was previously investigated in the 123 trial in which 222 patients, after reaching maximal N2O cooling flow, were randomized into short (2 × 1 min), medium (2 × 2 min), and long (2 × 3 min) cryoenergy durations, demonstrating that the shorter application time may impair the rate of sustained isolation of left side PVs, despite reducing the risk of PNP.

The plateau reached by the lesion at 180 s, as demonstrated in preclinical studies, fully explains the aforementioned results. Therefore, extending the exposure time beyond this point does not lead to any further increase in the size or volume of the lesion (37, 38); conversely, reducing the freeze duration can result in non-transmural lesions and consequently facilitate vein electrical reconnection and late arrhythmia recurrence. In clinical practice, when PNP occurs the cryoenergy application is immediately aborted to avoid further unwanted damage to the nerve. In our study, in the PNP+ group, we observed a median freeze duration of 134.0 [122.75, 150.75] sec and 133.5 [118.0, 149.5] sec for RIPV and RSPV respectively and additionally applications in the same PVs were not performed as electrical isolation had already been achieved in all cases. It is likely for the reasons discussed above that occurrence of PNP resulted to be an important independent predictor of AT/AF relapses in our study. The analysis of repeat procedures displayed how PNP+ patients were more than 8 times as likely to develop a right-sided PV reconnection compared to the PNP- group [OR 8.44; 95% CI (1.37; 52.06)]. Moreover, patients of the PNP+ group had a RMST 3.29 months shorter than their counterparts [95% CI (−5.11, −1.46), *p* < 0.001] given the optimal covariate balance provided by the PSM.

It is also noteworthy that, although most RIPV-related PNPs are transient and resolve during the procedure, in cases where PNP persists, the isolation of the RSPV—if electrically active—requires alternative ablation techniques. In our study, RF energy was used more frequently in the PNP+ group compared to the PNP- group, showing a trend toward statistical significance (*p* = 0.06).

To conclude, although infrequent, PNP during CB-A is not negligible and remains the most common procedure-related complication, with the potential to compromise long-term efficacy. Therefore, close intra-procedural monitoring of PN function is crucial to minimize the risk of permanent injury.

Both the diaphragmatic compound motor action potential (CMAP) and the Negrar Enhanced Diaphragmatic Motor Action Potential (NeedMap) techniques have proven effective in monitoring PN activity, with the goal of preventing cryothermal damage. A ≥30% reduction in CMAP amplitude has been identified as a reliable threshold for early detection of impending nerve injury. In a seminal study conducted on mongrel dogs, this criterion presaged diaphragmatic paralysis, as detected by abdominal palpation, by 31 ± 23 s (39–41).

Recently, Marinelli et al. compared CMAP and NeedMap in 150 patients, 15 of whom developed PN threat. NeedMap showed superior diagnostic performance, consistently reaching a 35% amplitude drop earlier than CMAP (41). Moreover, it demonstrated higher sensitivity (61% vs. 38%), specificity (86% vs. 76%), and overall diagnostic accuracy [AUC 0.806, 95% CI (0.68–0.92) vs. AUC 0.710, 95% CI (0.56–0.85)], with a combined sensitivity+ specificity index of 1.47 vs. 1.14.

Therefore, these methods, together with the use of shorter CB-A protocol for right-sided veins (e.g., 120 s × 2) may lead to a further reduction or even abolition in the occurrence rate of this complication.

Faster cooling rates typically correlates with better balloon contact and effective lesion formation (42–44). Although it did not appeared to be linked with a higher risk of developing PNP, we reported a faster first minute temperature drop for both RIPV and RSPV in the PNP+ group compared to the counterpart (*p* = 0.03 and *p* = 0.04, respectively). Later these veins showed paradoxically higher reconnection rates in the PNP+ group. This apparent contradiction may be explained by the premature interruption of energy delivery due to PNP, which likely prevented full lesion formation and compromised long-term durability despite favorable initial parameters.

With regard to other predictors of arrhythmia recurrence, our multivariable Cox regression model detected larger LA diameter, and need of ECV at the end of CB-A as independent predictors of AT/AF relapses during the follow-up.

Several previous studies have already shown that the occurrence and development of AF are closely related to atrial remodeling, which includes neural, electrical and structural remodeling (45–48). Structural remodeling of the atria, with dilation and fibrosis, is a hallmark of AF. These changes create a substrate conducive to the formation and maintenance of re-entrant circuits. Specifically, atrial enlargement allows for multiple re-entrant circuits to coexist, while fibrosis leads to conduction heterogeneity and prolonged refractoriness (49, 50). Moreover, atrial remodeling may lead to an increase in non-PV AF triggers (23). Since catheter ablation often focuses solely on the pulmonary veins without addressing other potential sources of arrhythmia, this may explain why left atrial enlargement can contribute to AT/AF relapses after ablation.

The presence of AF after a successful electrical isolation of all the PVs might reflect a higher degree of electrical and structural remodelling leading to arrhythmia perpetuation (51), and was already reported by our group as a potential predictor of AT/AF recurrence after ablation (21).

## 5 Limitations

The current study is a retrospective analysis involving typical limitations and epidemiological disadvantages. Moreover, the study was conducted exclusively using the Medtronic cryoballoon system; therefore, the same conclusions may not be generalizable to patients treated with other cryoballon technologies.

Although RF ablation could also further damage the PN in very rare cases, all the 5 patients in which RSPV was electrically active after RIPV-related persistent PNP, RF was used to isolate the veins, since pulse-field ablation technology was not available at that time in our centers.

With regard to follow-up, our study did not include standard implantation of loop recorder neither 7-days ECG-Holter, limiting our capacity to detect arrhythmia recurrences. Similarly, the complication rate may have been underestimated since none of our centers systematically performed esophagogastroduodenoscopy (EGDS) and heart CT-scan post-PVI to asymptomatic patients to look for silent esophageal wall injury or PV stenosis.

## 6 Conclusions

This study demonstrates that PNP during CB-A is associated with a lower success rate, higher risk of AT/AF recurrence after CB-A and a trend towards significance for RF touch-up to complete PVI. Therefore the routine adoption of cryoapplication protocols shorter than the standard 180 s or 240 s for right-sided PVs along with the use of instruments capable of promptly detecting reductions in phrenic nerve activity, may be advisable, to optimize procedural safety and preserve the use of cryoballon-ablation in the future.

## Data Availability

The datasets presented in this article are not readily available because they contain sensitive patient information. Requests to access the datasets should be directed to the corresponding author.
