# Supplementary material for: Phrenic nerve palsy during cryoballoon ablation of atrial fibrillation: a minor complication or a wolf in sheep's clothing? Insights on late arrhythmia recurrences from a propensity score-matched analysis
Source: Front Cardiovasc Med. 2025 Sep 29;12:1650358. doi: 10.3389/fcvm.2025.1650358 (PMC12515864; doi:10.3389/fcvm.2025.1650358)
Supplement: Supplementary file 1 [file Datasheet1.pdf]

SUPPLEMENTARY MATERIAL 1 – PROPENSITY SCORE MATCHING QUALITY ASSESSMENT

1- Love plot

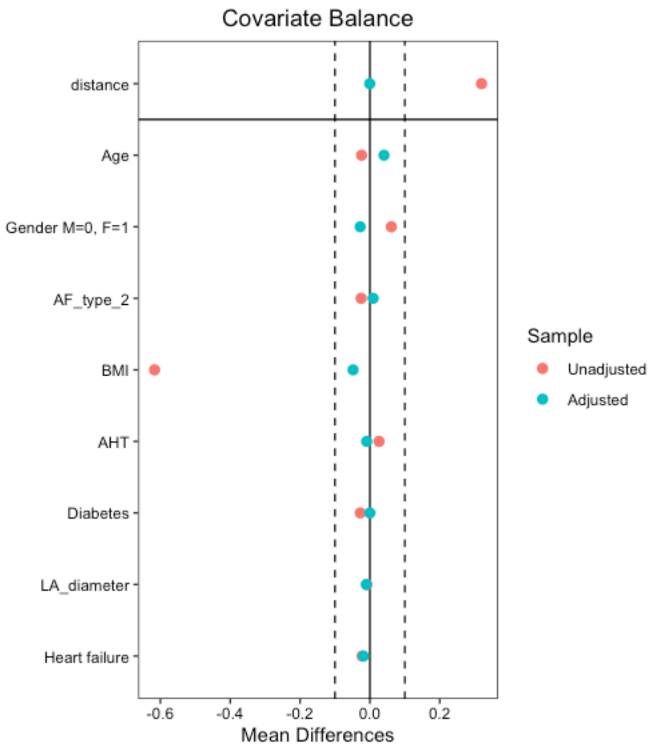

The love plot illustrates the effectiveness of propensity score matching in balancing key covariates between treatment and control group. Before matching, unadjusted differences (orange points) were significant for variables like BMI, gender, and age. However, after matching, adjusted differences (blue points) were substantially reduced and fell below the commonly accepted threshold of 0.1. This indicates that the matching model successfully controlled for confounding factors, ensuring comparability between the groups.

2- Standardized mean difference

```
Balance Measures
                    Type Diff.Adj    M.Threshold
distance        Distance  -0.0004 Balanced, <0.1
Age               Contin.  0.0404 Balanced, <0.1
Gender M=0, F=1   Binary  -0.0278 Balanced, <0.1
AF_type_2         Binary   0.0093 Balanced, <0.1
BMI              Contin.  -0.0482 Balanced, <0.1
AHT               Binary  -0.0093 Balanced, <0.1
Diabetes          Binary   0.0000 Balanced, <0.1
LA_diameter      Contin.  -0.0101 Balanced, <0.1
Heart failure     Binary  -0.0185 Balanced, <0.1

Balance tally for mean differences
                    count
Balanced, <0.1          9
Not Balanced, >0.1      0

Variable with the greatest mean difference
 Variable Diff.Adj    M.Threshold
      BMI  -0.0482 Balanced, <0.1

Sample sizes
          Control Treated
All          1440     108
Matched       108     108
Unmatched    1332       0
```

The standardized mean difference (SMD) analysis demonstrates excellent covariate balance between the treatment and control groups after propensity score matching. All variables exhibited adjusted differences below the critical threshold of 0.1, indicating effective control for confounding factors. The largest SMD was for BMI (-0.0482), yet it remained well within the acceptable range. The balance tally further confirms the effectiveness of the matching process. These findings suggest that the subsequent analysis can be considered reliable and not influenced by baseline differences between the groups.

3- Variance ratio

Balance Measures

	Type	Diff.Un	Diff.Adj
distance	Distance	0.3197	-0.0004
Age	Contin.	-0.0238	0.0404
Gender M=0, F=1	Binary	0.0613	-0.0278
AF_type_2	Binary	-0.0250	0.0093
BMI	Contin.	-0.6167	-0.0482
AHT	Binary	0.0262	-0.0093
Diabetes	Binary	-0.0275	0.0000
LA_diameter	Contin.	-0.0087	-0.0101
Heart failure	Binary	-0.0225	-0.0185

Sample sizes

	Control	Treated
All	1440	108
Matched	108	108
Unmatched	1332	0

The variance ratio table shows the differences between control and treated groups before (Diff.Un) and after (Diff.Adj) propensity score matching. Initially, there are noticeable imbalances in variables like BMI (0.6167), gender (0.0613), and AF type 2 (0.0250). After matching, these differences are significantly minimized, with values like BMI reduced to 0.0482 and gender to -0.0278, demonstrating improved balance between the groups. The total sample consists of 1,440 controls and 108 treated subjects, with 108 matched pairs, indicating that the matching process successfully balanced the covariates across treated and control groups, enhancing the validity of subsequent analyses.
