# Supplementary material for: Phrenic nerve palsy during cryoballoon ablation of atrial fibrillation: a minor complication or a wolf in sheep's clothing? Insights on late arrhythmia recurrences from a propensity score-matched analysis
Source: Front Cardiovasc Med. 2025 Sep 29;12:1650358. doi: 10.3389/fcvm.2025.1650358 (PMC12515864; doi:10.3389/fcvm.2025.1650358)
Supplement: Supplementary file 2 [file Datasheet2.pdf]

SUPPLEMENTARY MATERIAL_2

Table_1. UNIVARIABLE COX REGRESSION ON BASELINE CHARACTERISTICS

Baseline characteristics	P	CI	HR
**Age**	0.29	0.99 – 1.03	1.01
**BMI**	0.87	0.94 – 1.06	1.00
**Gender**	0.76	0.57 – 1-50	0.93
**Paroxysmal AF**	0.13	0.41 – 1.12	0.67
**Persistent AF**	0.24	0.81 – 2.25	1.36
**AF duration**	0.94	0.99 – 1.00	1.00
**LA diameter**	0.003	1.02 – 1.09	1.02
**LVEF**	0.42	0.96 – 1.02	0.42
**Heart failure**	0.67	0.49 – 3.03	1.22
**Dilated cardiomyopathy**	-	-	-
**Coronary artery disease**	0.77	0.41 – 1.95	0.89
**Arterial hypertension**	0.67	0.69 – 1.79	1.11
**Diabetes mellitus**	0.34	0.18 – 1.81	0.57
**COPD**	-	-	-
**CKD**	0.02	1.11 – 3.87	2.71
**Creatinine**	0.02	1.47 – 65.93	9.85
**Prior Stroke/TIA**	0.38	0.63 – 3.39	0.38
**PM**	0.26	0.65 – 4.95	1.80
**ICD**	0.42	0.49 – 5.43	0.42
**CHA_2DS_2VASc score**	0.45	0.92 – 1.23	1.06
**N° of ECV before PVI**	0.30	0.85 – 1.71	1.20
**N° of ADDs tested**	0.81	0.73 – 1.28	0.97
**Ic class AADs**	0.42	0.49 – 1.35	0.81
**Beta blockers**	0.48	0.52 – 1.36	0.84
**Amiodarone**	0.13	0.85 – 3.47	1.72
**Sotalol**	0.57	0.59 – 2.61	1.24

The table presents the results of the univariate Cox regression analysis aimed at identifying baseline patient characteristics associated with AT/AF recurrences after cryoballoon ablation (CB-A). The P column indicates the p-value of the test, reflecting the statistical significance of each variable. The HR (Hazard Ratio) column represents the relative risk of recurrence associated with each characteristic, and the CI (Confidence Interval) column provides the 95% confidence interval for the hazard ratio, showing the range within which the true hazard ratio is likely to fall. Variables with a p-value below 0.05, such as LA diameter, CKD, and creatinine, are underlined in yellow. BMI = body mass index, AF = atrial fibrillation, LA = left atrium, LVEF = left ventricular ejection fraction, COPD = chronic obstructive pulmonary disease, CKD = chronic kidney disease, eGFR = estimated glomerular filtration rate (according to the Cockcroft-Gault formula), TIA = transient ischemic attack, CHA2DS2VASc = congestive heart failure, hypertension, age, diabetes mellitus; stroke/TIA; Vascular arterial disease; sex category (female), AAD = antiarrhythmic drugs

Table_2. UNIVARIABLE COX REGRESSION ON PROCEDURAL CHARACTERISTICS

Procedural Characteristics	P	CI	HR
Procedure duration	0.47	0.99 – 1.01	1.00
Fluoroscopic exposure	0.420	0.98 – 1.05	1.05
PVs anatomical variants			
Left common ostium	0.13	0.13 – 1.30	0.41
Right middle pulmonary vein	0.86	0.44 – 2.71	1.09
AF at the beginning of CB-A	0.17	0.82 – 3.22	1.62
SR restoration during PVI	0.53	0.07 – 3.83	0.53
ECV at the end	0.02	1.11 – 3.15	1.87
Phrenic nerve palsy	0.002	1.34 – 3.67	2.22
LSPV			
Number of freezes	0.12	0.27 – 1.15	0.55
Temperature at 60 sec	0.74	0.95 – 1.03	0.99
Minimum temperature	0.40	0.93 – 1.03	0.40
Total freeze duration	0.11	0.99 – 1.09	1.00
LIPV			
Number of freezes	0.08	0.33 – 1.07	0.590
Temperature at 60 sec	0.59	0.95 – 1.09	1.02
Minimum temperature	0.89	0.95 – 1.05	0.89
Total freeze duration	0.89	0.96 – 1.04	1.00
LCO			
Number of freezes	-	-	-
Temperature at 60 sec	0.48	0.60 – 3.01	1.34
Minimum temperature	0.32	0.54 – 6.89	1.92
Total freeze duration	0.82	0.87 – 1.19	1.02
RIPV			
Number of freezes	0.11	0.12 – 1.23	0.26
Temperature at 60 sec	1.00	0.94 – 1.06	1.00
Minimum temperature	0.82	0.97 – 1.02	1.00
Total freeze duration	0.03	0.99 – 1.00	1.00
RSPV			
Number of freezes	0.11	0.28 – 1.14	0.56
Temperature at 60 sec	0.55	0.95 – 1.10	1.02
Minimum temperature	0.97	0.97 – 1.03	1.00
Total Freeze duration	0.003	0.99 – 1.00	0.99

The table presents the results of the univariate Cox regression analysis aimed at identifying procedural characteristics associated with AT/AF recurrences after cryoballoon ablation (CB-A). As for the precious table the P column indicates the p-value of the test, the HR (Hazard Ratio) column represents the relative risk of recurrence associated with each characteristic, and the CI (Confidence Interval) column provides the 95% confidence interval for the HR. Variables with a p-value below 0.05, such as ECV at the end, phrenic nerve palsy, RSPV and RIPV total freeze duration are underlined in yellow. CB-A = Cryoballoon ablation, LSPV = left superior pulmonary vein, LIPV = left inferior pulmonary vein, LCO = left common ostium, RIPV = right inferior pulmonary vein, RSPV = right superior pulmonary vein, RMPV = right middle pulmonary vein.

Table_3

C-index = 0.723

Schoenfield Test

	Chi-squared	df	P
**Total number of LIPV freezes**	0.788	1	0.37
**Left atrial diameter**	0.656	1	0.42
**Chronic kidney disease**	0.012	1	0.91
**Need of ECV after PVI**	0.008	1	0.93
**Phrenic nerve palsy**	2.474	1	0.12
**Global**	4.787	5	0.44

The table presents the results of the Shoenfield residuals used to evaluate the proportional hazards assumption of the multivariable Cox regression analysis. The Schoenfeld residuals test confirmed that the proportional hazards assumption was not violated for the variables included in the multivariate Cox model, with all p-values > 0.05 and a global test p-value of 0.44. On the top, the C-index of 0.723 indicates a good predictive performance of the model. LIPV = left inferior pulmonary vein, ECV = electrical cardioversion, PVI = pulmonary vein isolation, P = p value, df = degree of freedom.
