# Supplementary material for: Phrenic nerve palsy during cryoballoon ablation of atrial fibrillation: a minor complication or a wolf in sheep's clothing? Insights on late arrhythmia recurrences from a propensity score-matched analysis
Source: Front Cardiovasc Med. 2025 Sep 29;12:1650358. doi: 10.3389/fcvm.2025.1650358 (PMC12515864; doi:10.3389/fcvm.2025.1650358)
Supplement: Supplementary file 3 [file Datasheet3.pdf]

SUPPLEMENTARY MATERIAL_3
Potential predictors of phrenic nerve palsy

Table_1_RIPV

Baseline and procedural characteristics	P	OR	HR
**Age**	0.48	0.99	0.96 -1.02
**BMI**	0.86	1.01	0.93 – 1.10
**Gender**	0.54	0.80	0.39 – 1.63
**Paroxysmal AF**	0.68	1.19	0.53 – 2.10
**Persistent AF**	0.58	0.79	0.35 – 1.80
**AF duration**	0.83	1.00	0.99 – 1.01
**LA diameter**	0.67	0.99	0.94 – 1.04
**LVEF**	0.99	1.00	0.99 – 1.01
**Heart failure**	0.59	1.38	0.43 – 4.45
**Dilated cardiomyopathy**	1.00	-	-
**Coronary artery disease**	0.22	0.39	0.09 – 1.75
**Arterial hypertension**	0.95	1.02	0.51 – 2.06
**Diabetes mellitus**	0.70	1.30	0.35 – 4.91
**COPD**	-	-	-
**CKD**	0.77	1.19	0.38 – 3.79
**Creatinine**	0.36	3.21	0.26 – 39.62
**Prior Stroke**	0.70	1.30	0.35 – 4.91
**PM**	0.22	2.44	0.58 – 10.24
**ICD**	0.58	1.59	0.31 – 8.21
**CHA_2DS_2VASc score**	0.48	0.92	0.73 – 1.16
**n. of ECV before CB-A**	0.55	0.82	0.43 – 1.55
**Procedure duration**	0.41	1.01	0.99 -1.02
**Fluoroscopy exposure**	0.72	1.01	0.97 – 1.05
**Presence of RMPV**	0.49	1.61	0.42 - 6.25
**Time to isolation**	0.33	0.98	0.95 – 1.02
**RIPV temperature at 60 sec**	0.07	0.93	0.85 – 1.01
**RIPV minimum temperature**	0.61	1.02	0.94 - 1.10

The table presents the results of the univariable binary logistic regression analysis aimed at identifying baseline patient characteristics associated with RIPV phrenic nerve palsy. The P column indicates the p-value of the test, reflecting the statistical significance of each variable. The OR (Odds Ratio) column represents the relative odds of recurrence associated with each characteristic, and the CI (Confidence Interval) column provides the 95% confidence interval for the odds ratio. BMI = body mass index, AF = atrial fibrillation, LA = left atrium, LVEF = left ventricular ejection fraction, COPD = chronic obstructive pulmonary disease, CKD = chronic kidney disease, eGFR = estimated glomerular filtration rate (according to the Cockcroft-Gault formula), TIA = transient ischemic attack, CHA2DS2VASc = congestive heart failure, hypertension, age, diabetes mellitus; stroke/TIA; Vascular arterial disease; sex category (female), ECV = electrical cardioversion, CB-A = cryoballoon ablation, RIPV = right inferior pulmonary vein

Table_1_RSPV

Baseline and procedural characteristics	P	OR	CI
**Age**	0.37	1.01	0.99 – 1.08
**BMI**	0.64	0.98	0.91 – 1.05
**Gender**	0.95	1.02	0.57 – 1.81
**Paroxysmal AF**	0.86	0.94	0.50 – 1.80
**Persistent AF**	0.54	1.22	0.65 – 2.29
**AF duration**	0.54	1.00	0.99 – 1.01
**LA diameter**	0.79	1.01	0.97 – 1.05
**LVEF**	0.67	1.01	0.97 – 1.06
**Heart failure**	0.34	0.57	0.18 – 1.80
**Dilated cardiomyopathy**	1.00	-	-
**Coronary artery disease**	0.72	0.84	0.33 - 2.14
**Arterial hypertension**	0.84	0.94	0.53 – 1.67
**Diabetes mellitus**	0.75	0.82	0.25 – 2.73
**COPD**	-	-	-
**CKD**	0.45	1.44	0.56 – 3.70
**Creatinine**	0.48	0.41	0.03 – 4.93
**Prior Stroke/TIA**	0.75	0.82	0.25 – 2.73
**PM**	0.52	0.59	0.12 – 2.93
**ICD**	0.65	0.69	0.13 – 3.49
**CHA_2DS_2VASc score**	0.83	1.02	0.85 – 1.23
**n. of ECV before CB-A**	0.06	1.55	0.98 – 2.43
**Procedure duration**	0.66	1.00	0.99 – 1.01
**Fluoroscopy exposure**	0.31	1.02	0.98 – 1.06
**Presence of RMPV**	0.58	0.69	0.18 – 0.97
**Time to isolation**	0.75	0.99	0.96 – 1.03
**RSPV temperature at 60 sec**	0.008	0.89	0.81 – 0.97
**RSPV minimum temperature**	0.25	0.96	0.91 - 1.03

The table presents the results of the univariable binary logistic regression analysis aimed at identifying baseline and procedural patient characteristics associated with RSPV phrenic nerve palsy. The P column indicates the p-value of the test. the OR (Odds Ratio) column represents the relative odds of recurrence associated with each characteristic, and the CI (Confidence Interval) column provides the 95% confidence interval for the odds ratio. BMI = body mass index, AF = atrial fibrillation, LA = left atrium, LVEF = left ventricular ejection fraction, COPD = chronic obstructive pulmonary disease, CKD = chronic kidney disease, eGFR = estimated glomerular filtration rate (according to the Cockcroft-Gault formula), TIA = transient ischemic attack, CHA2DS2VASc = congestive heart failure, hypertension, age, diabetes mellitus; stroke/TIA; Vascular arterial disease; sex category (female), ECV = electrical cardioversion, CB-A = cryoballoon ablation, RSPV = right superior pulmonary vein

Table_3

	P	OR	CI
**RSPV temperature at 60 sec**	0.88	0.98	0.80 – 1.22
**n. of ECV before CB-A**	0.87	1.08	0.43 – 2.70

The table presents the results of the multivariable binary logistic regression analysis aimed at identifying baseline and procedural patient characteristics associated with RSPV phrenic nerve palsy. As indicated by the p value of the test, none of the two variables appeared to be linked with phrenic injury. p = P value, OR = odds ratio, CI = confidence interval. RSPV = right superior pulmonary vein, ECV = electrical cardioversion, CB-A = cryoballoon ablation
